# New Appliances and Things Medical

**Published:** 1900-03-03

**Authors:** 


### NEW APPLIANCES AND THINGS MEDICAL.

[We shall be glad to receive, at our Office, 28 & 29, Southampton Street, Strand, London, W.C., from the manufacturers, specimens of all new preparations and appliances which may be brought out from time to time.]

### INVALID FURNITURE.

Red Cross aid may be said to be one of the watchwords of the war, and in consequence the makers of invalid furniture have brought forward some excellent examples of portable contrivances for the convenience of the sick and wounded. Amongst others the handy little bed-table, introduced by Messrs. Farmer, Lane, and Co., of 77 and 79, New Oxford Street, is worthy of notice. Its first appearance is that of a light tray of polished wood; four slender legs are screwed in, and the tray becomes a bed-table; a section is raised in the middle, and a rest fixed before it, and the invalid has at his command an adjustable reading-stand. The prices are from 7s. 6d. Another requisite for invalid comfort is the transport lounge; it is made in strong cane, the back is adjustable, and a leg rest slides in and out under the seat as required. The cheap "Merlin" chairs and bed-rests made by this firm are strong, light, easily disinfected, and moderate in price. The Government has placed a large order for the bed-rest for use in the home military hospitals.

### WINES FOR INVALIDS.

(Messrs. Innes, Smith, and Co., 83, High Street, Birmingham.)

We have received samples of various wines from the above firm with a view to testing their suitability for invalids and convalescents. The list includes a very fine dry champagne (Jules Delporte) at 75s. per dozen. This is undoubtedly a very fine wine, free from the acidulous and saccharine products of indifferent fermentation which are so harmful to those of gouty or rheumatic tendency. Of the red wines there are examples of French, Hungarian, Portuguese, and Australian manufacture. Of these the Hungarian, at 26s. per dozen, is unmistakably a pure wine, is very clean, and is well suited to those who cannot digest the richer wines, such as the Australian and French (No. 2). These two latter are well fitted for cases of anæmia and debility, especially the Australian, which possesses ferruginous properties. The Moselle (No. 6) at 26s. is a wonderfully cheap wine considering its class; it has a fine aromatic flavour, and will be much appreciated by those who fancy the white wines of German production.

### MILKINE TABLETS AND POWDER.

(Elgin Milkine Company, Elgin, Ill., U.S.A. Agents: Gilbert, Kimpton, and Co., 19, St. Dunstan's Hill, E.C.)

Milkine is a desiccated food prepared from milk, beef, and farinaceous elements. In the process of manufacture the farinaceous ingredients are malted and converted into dextrins and sugars. The food is a complete food, and easily digested, fitted for invalids or infants, and in the tablet form most convenient for travellers and the like, who must of necessity carry a concentrated and sustaining food in a small compass. As a temporary food for infants who are unable to digest ordinary cow's milk it has had excellent results. It must be remembered, however, that if intended as a complete infant food it is deficient in fat.

### GAMBIER'S ANTI-ASTHMATIC.

(London Agents: Gilbert, Kimpton, and Co., 19, St. Dunstan's Hill, E.C.)

This powder, which can be smoked and inhaled in the form of a cigarette or simply burnt in the room, has undoubted antispasmodic influences on the bronchial tubes, and in cases of asthma gives almost instantaneous relief. It is a useful combination of the vegetable substances which experience shows are most efficacious in the treatment of this complaint. Gambier's powder is certainly a skilfully combined preparation, and may be given a trial whenever the ordinary routine methods have failed.

